# Localized delivery of compounds into articular cartilage by using high-intensity focused ultrasound

**DOI:** 10.1038/s41598-019-52012-z

**Published:** 2019-11-04

**Authors:** Heikki J. Nieminen, Eetu Lampsijärvi, Gonçalo Barreto, Mikko A. J. Finnilä, Ari Salmi, Anu J. Airaksinen, Kari K. Eklund, Simo Saarakkala, Kenneth P. H. Pritzker, Edward Hæggström

**Affiliations:** 1 Electronics Research Laboratory, Department of Physics, University of Helsinki, Helsinki, Finland; 2 Department of Neuroscience and Biomedical Engineering, Aalto University, Espoo, Finland; 3 Research Group of Medical Imaging, Physics and Technology, Faculty of Medicine, University of Oulu, Oulu, Finland; 4 Department of Medicine, University of Helsinki, Helsinki, Finland; 5 Orton Orthopaedic Hospital and Research Institute, Invalid Foundation, Helsinki, Finland; 6 Department of Chemistry - Radiochemistry, University of Helsinki, Helsinki, Finland; 7 Department of Rheumatology, University of Helsinki and Helsinki University Hospital, Helsinki, Finland; 8 Department of Diagnostic Radiology, Oulu University Hospital, Oulu, Finland; 9 Department of Laboratory Medicine and Pathobiology, University of Toronto and Mount Sinai Hospital, Toronto, Canada

**Keywords:** Cartilage, Acoustics, Optical physics

## Abstract

Localized delivery of drugs into an osteoarthritic cartilaginous lesion does not yet exist, which limits pharmaceutical management of osteoarthritis (OA). High-intensity focused ultrasound (HIFU) provides a means to actuate matter from a distance in a non-destructive way. In this study, we aimed to deliver methylene blue locally into bovine articular cartilage *in vitro*. HIFU-treated samples (*n* = 10) were immersed in a methylene blue (MB) solution during sonication (*f* = 2.16 MHz, peak-positive-pressure = 3.5 MPa, mechanical index = 1.8, pulse repetition frequency = 3.0 kHz, cycles per burst: 50, duty cycle: 7%). Adjacent control 1 tissue (*n* = 10) was first pre-treated with HIFU followed by immersion into MB; adjacent control 2 tissue (*n* = 10) was immersed in MB without ultrasound exposure. The MB content was higher (*p* < 0.05) in HIFU-treated samples all the way to a depth of 600 µm from AC surface when compared to controls. Chondrocyte viability and RNA expression levels associated with cartilage degeneration were not different in HIFU-treated samples when compared to controls (*p* > 0.05). To conclude, HIFU delivers molecules into articular cartilage without major short-term concerns about safety. The method is a candidate for a future approach for managing OA.

## Introduction

Osteoarthritis (OA) is one of the leading causes of disability worldwide. Pathogenetically OA exhibits changes in osteochondral tissue, *i.e*. structural and compositional changes in articular cartilage (AC) and underlying bone^1,2^ The OA lesions can involve only limited areas of the cartilage^3^. However, methods to deliver therapeutic compounds into such lesions are still mostly unavailable. The active pharmaceutical ingredient (API) can be delivered systemically^4^, *e.g*. orally or locally as intra-articular injections. In both approaches, the API may not reach the target cartilage tissue unless excessive doses, which may be toxic are administered. At present, clinical methods to deliver a therapeutic compounds directly into a local OA cartilage lesion do not exist.

High intensity ultrasound (HIU) provides a way to manipulate tissue from a distance in a non-invasive manner^5^. A traveling ultrasound wave carries momentum, which is converted into a force *e.g*. at medium interfaces or inside objects^6^, where the momentum of the wave is changed. *E.g*. when sound energy is absorbed into a medium, reflected from an acoustic interface or scattered from a particle or void, an acoustic radiation force is exerted^7–9^. When the ultrasound energy is absorbed into liquid, acoustic streaming may form^7,10^. Importantly, HIU can palpate a material or translate particles and gas voids non-destructively^6,11,12^ from a distance, *e.g*. through the skin within the body. Interaction with micro-bubbles, *i.e*. during cavitation, can induce micro-streams or shear forces near the bubbles^12^, which can lead to tissue permeabilization^13,14^ or if excess, to tissue emulsification^15^. If the time-averaged ultrasound intensity is high, the absorbed ultrasound energy appears as heating, which can induce tissue edema^16^ or thermal necrosis^17^. Because HIU can influence tissue in so many ways, it can provide a way to address targeted and localized release, deposition, and translation of drugs for therapeutic purposes.

We previously demonstrated that ultrasound can deliver molecules into AC non-destructively^18–21^. MHz high-intensity focused ultrasound (HIFU, *i.e*. HIU that is generated by geometrically focusing the ultrasound to a focal spot) (1.14 MHz) delivered 2.8 kDa-size molecules into AC to a depth of 700 to 800 µm in 2.5 hours without inducing histologically discernible damage^19,20^. In a recent study, we demonstrated that laser ultrasound -induced shock waves (center frequency 3 MHz) were capable of delivering 320 Da molecules to a depth of 600 µm in a clinically relevant timeframe of 11 minutes^21^. Importantly this induced no structural damage to the AC and neither affected AC cell (chondrocyte) viability nor their RNA expression^21^. Therefore, it seems that HIU provides a means to deliver molecules whose size is equivalent to that of drugs into AC without significant short-term adverse effects to AC.

Capability of HIFU to deliver small molecules into AC, while considering the impact on viability and the cellular response to HIFU exposure has not been demonstrated previously. In this study, we investigate if HIFU can deliver a 320 Da sized molecule into AC without affecting the short-term viability or RNA expression of chondrocytes.

## Results

**Figure 1:**
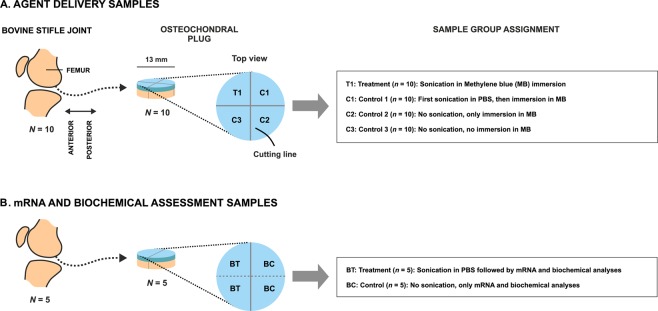
Sample preparation workflow and group assignment for agent delivery assessment (**A**) and mRNA and biochemical assessment samples (**B**).

**Figure 2:**
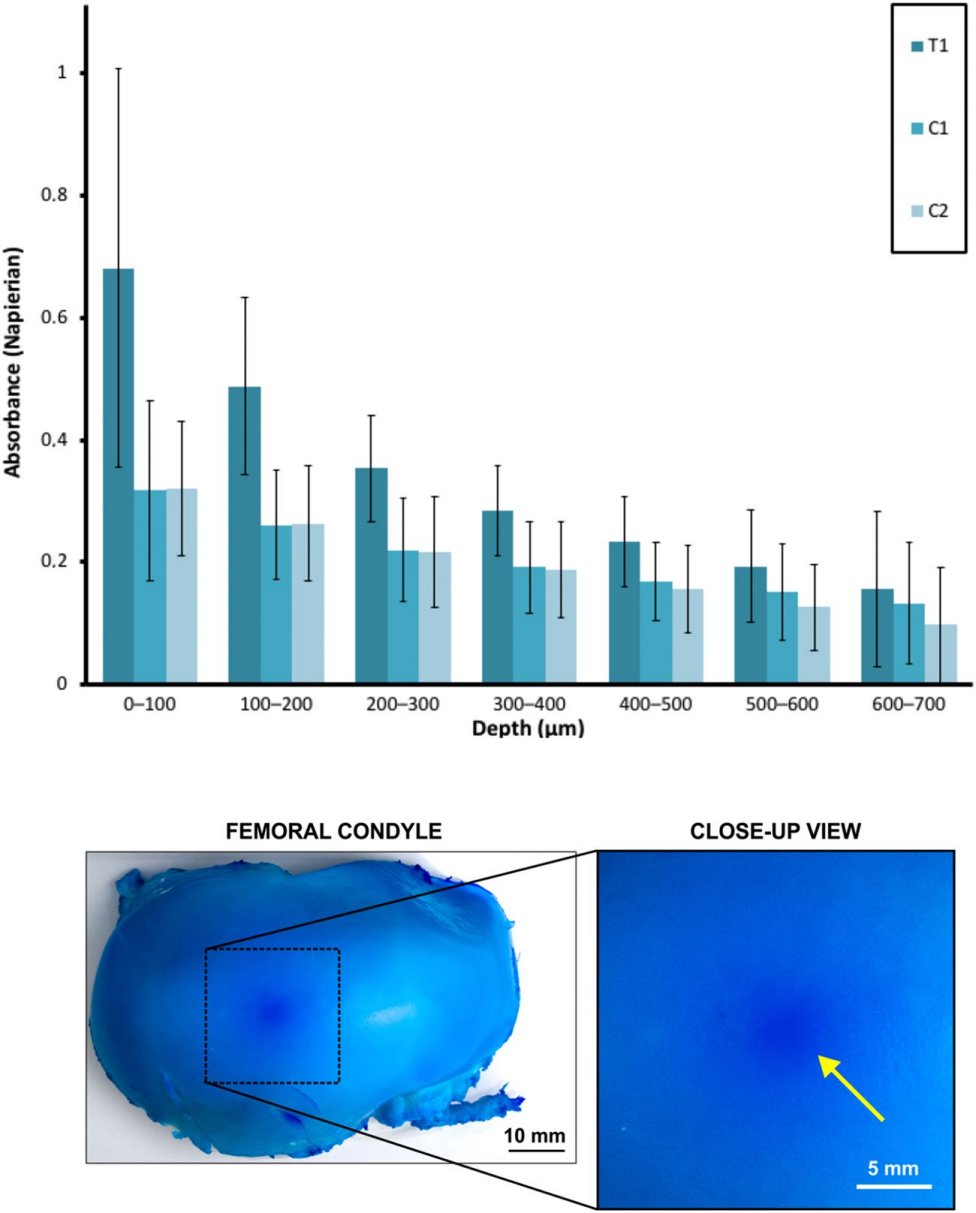
**Top:** Napierian absorbance in treated samples (T1) and controls 1 and 2 (C1 and C2, respectively) (mean ± 95% confidence intervals) as a function of depth from AC surface compensated with absorbance in C3. Higher levels of absorbance in T1 (determined at the absorbance peak of methylene blue, *i.e*. 657 nm) compared to controls suggest that HIFU enabled enhanced delivery of methylene blue in a clinically relevant time frame of 15 minutes. **Bottom:** Laterally localized contrast enhancement with a size 3–4 mm in diameter was observed in a bovine femoral condyle after HIFU treatment while the sample was immersed in PBS with methylene blue.

**Figure 3:**
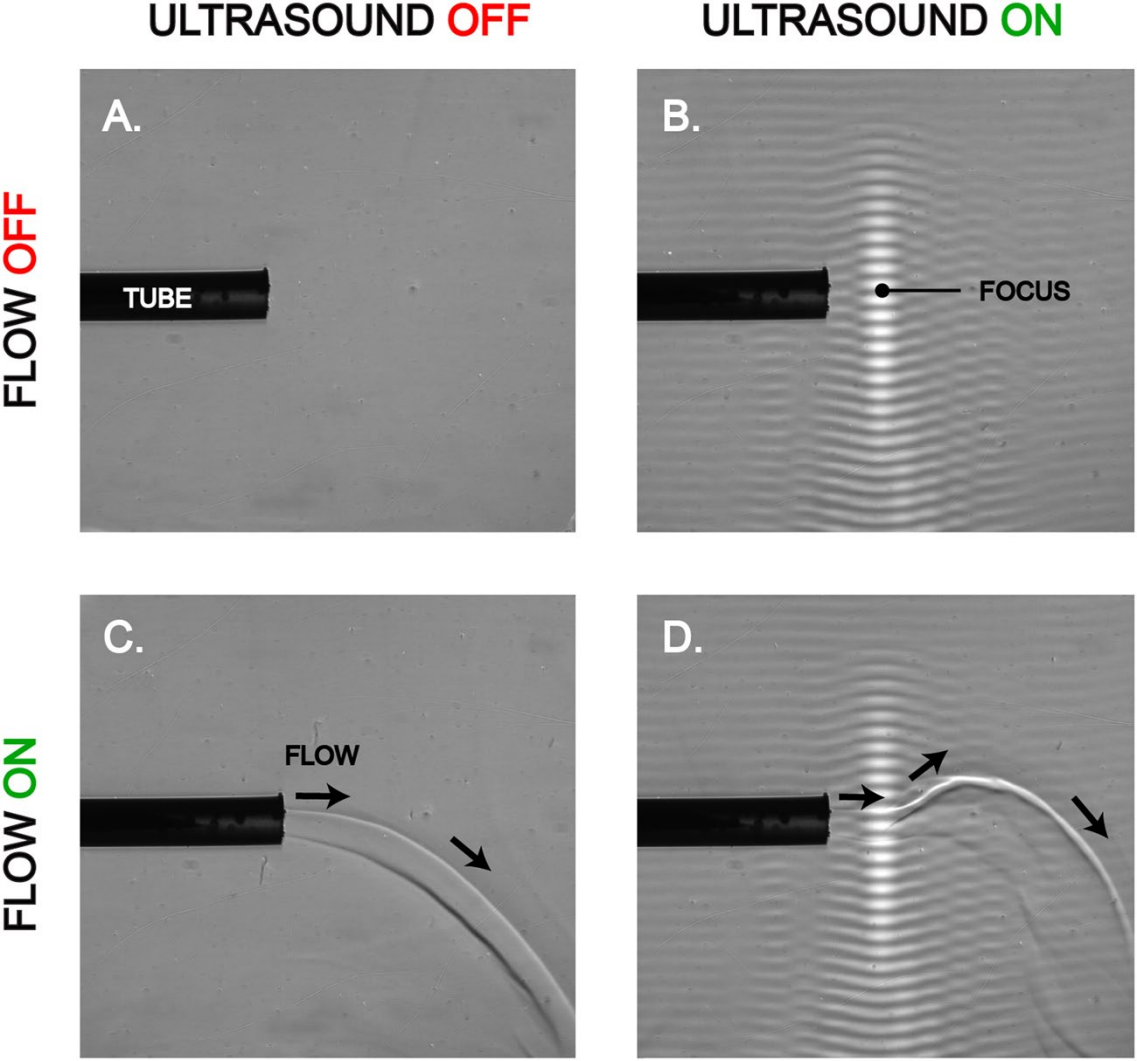
To demonstrate acoustic streaming at the acoustic focus and to simultaneously visualize the acoustic beam, a custom-made Schlieren imaging system was applied. A horizontal flow of cooled water was applied through a nozzle into a water bath to visualize acoustic streaming. The sound is propagating upwards. (**A**) Ultrasound on, horizontal flow off. (**B**) Ultrasound on, horizontal flow off. (**C**) Ultrasound off, horizontal flow on. (**D**) Ultrasound on, horizontal flow on. When ultrasound is applied simultaneously with horizontal flow, the direction of liquid flow is changing due to upward acoustic streaming.

Bovine osteochondral samples that were immersed in a contrast agent (methylene blue, MB) while being sonicated (T1 and femoral condyle) (Fig. 1, Table 1) were colored with pronounced blue intensity compared to adjacent tissue. This laterally localized contrast was 3–4 mm in diameter, exemplified in a full bovine femoral condyle (Fig. 2, Table 1). The depth-wise light absorbance at optical wavelength 657 nm, matching with the MB absorbance peak near 665 nm, was statistically different (*p* < 0.05) in treated samples T1 up to a depth of 600 µm from the AC surface as compared to sonicated and non-sonicated controls C1 and C2, respectively (Fig. 2, Table 1). Light absorbance in C1 and C2 was not statistically significantly different (*p* > 0.05) except at the depth of 500–700 µm (*p* < 0.05; Fig. 2). The HIFU-induced delivery of methylene blue into AC and into the full femoral condyle was thus achieved in a clinically relevant time frame of 15 minutes. The sample temperatures before sonication were 26.1 ± 1.1 **°**C (mean ± 95% CI; *N* = 5) and 26.3 ± 1.2 **°**C (*N* = 5) for T1 and C1, respectively; the end temperatures were 28.7 ± 2.1 **°**C (*N* = 5) and 28.8 ± 2.6 **°**C (*N* = 5) after sonication, respectively. The start and end temperatures for C2 were 29.0 ± 0.9 **°**C and 29.1 ± 0.8 **°**C (*N* = 5), respectively. The worst case estimates for thermal dose in *in vivo* situation with temperature elevations observed in this study were 0.19 ± 0.30 and 0.17 ± 0.26 CEM43 (*N* = 5) for T1 and C1, respectively. Acoustic streaming was confirmed under Schlieren imaging (Fig. 3).

**Table 1:** Summary of experimental procedures by sample group.

Sample	HIFU exposure during PBS immersion	HIFU exposure during MB immersion	Immersion in MB	Characterizing technique
Treatment, T1 (*n* = 10)	No	Yes	No	LM
Control 1, C1 (*n* = 10)*	Yes	No	Yes	LM
Control 2, C2 (*n* = 10)	No	No	Yes	LM
Control 3, C3 (*n* = 10)	No	No	No	LM
mRNA and viability assessment, treatment, BT (*n* = 5)	Yes	No	No	mRNA, viability
mRNA and viability assessment, control, BT (*n* = 5)	No	No	No	mRNA, viability
Complete femoral condyle	No	Yes	Yes (adjacent tissue)	Photograph

The procedures for each sample were applied chronologically from the far-left column towards the far-right column. The protocols were partially adapted from Nieminen *et al*.^21^.

*The time between sonication and MB immersion was <20 min.

HIFU: high-intensity focused ultrasound.

PBS: phosphate-buffered saline.

MB: methylene blue (0.005% w/v) in PBS.

LM: light microscopy in transmission mode at peak wavelength 657 nm.

mRNA: mRNA analysis.

**Figure 4:**
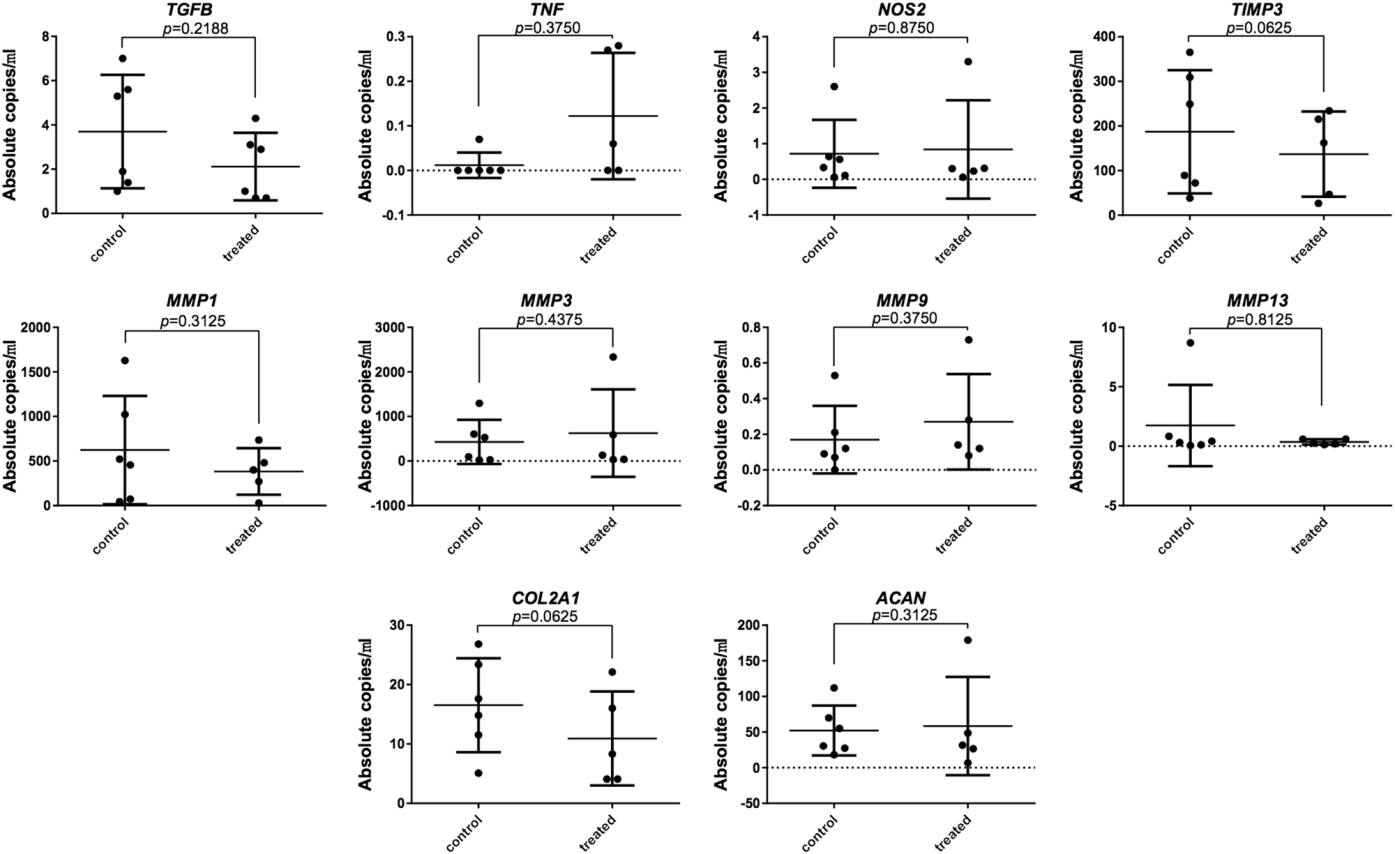
Effect of HIFU on the chondrocyte expression of catabolic, inflammatory, and anabolic markers dysregulated in OA as determined from mRNA (mean ± 95% confidence intervals; samples were measured as technical duplicates and averaged). The mRNA levels were not modified by HIFU (*p* > 0.05, Wilcoxon) suggesting that there are no major HIFU-induced adverse effects on the chondrocytes in the short-term.

**Figure 5:**
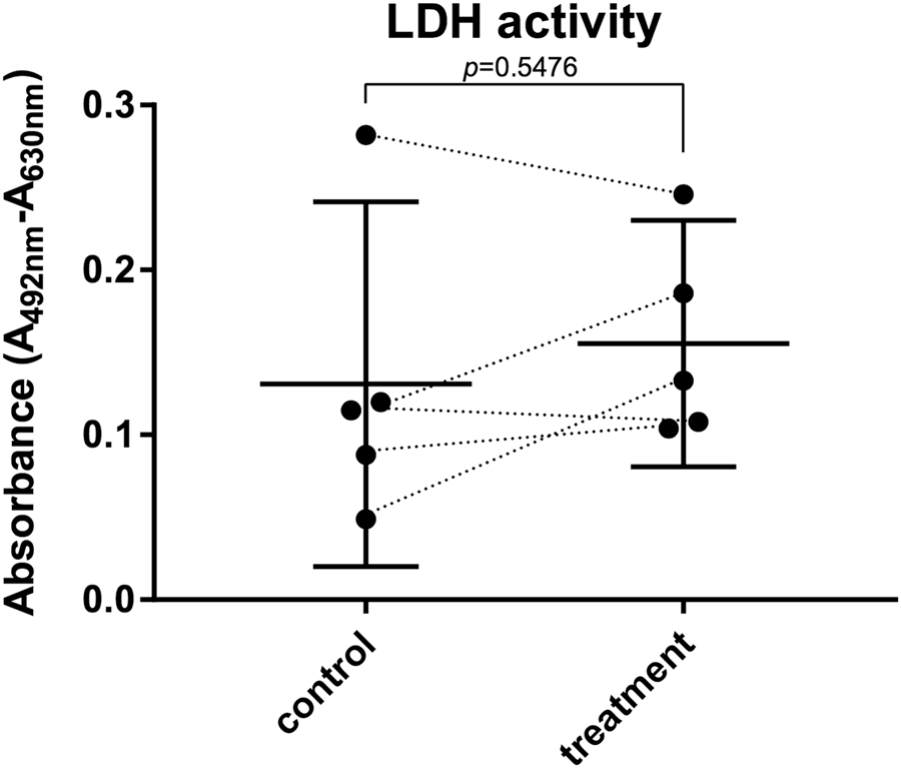
The LDH activity of treated samples was not statistically different (*p* = 0.5476) suggesting that viability of chondrocytes was not affected by HIFU.

The mRNA expression levels in BT (treatment samples, mRNA and biochemical assessment) were not statistically different (*p* > 0.05) from the mRNA expression levels in BC (control samples, mRNA and biochemical assessment) (Fig. 4). The assessment of viability as reflected by LDH levels of media of samples subjected to HIFU (BT) were not statistically different (*p* = 0.5746) as compared to the non-sonicated adjacent control (BC) (Fig. 5).

## Discussion

The light absorbance (657 nm) in T1 was in superficial AC approximately twice the light absorbance in adjacent controls C1 and C2. As compared to controls, the absorbance of T1 was statistically greater (*p* < 0.05) up to a depth of 600 µm from the AC surface. This result suggests that HIFU contributed to MB delivery. Laterally, the area with visible delivery was also confined, suggesting that HIFU-induced delivery is localized to a volume with a width of 3–4 mm and up to a depth of 600 µm (Fig. 2). In our earlier studies, the lateral width of the ultrasound-delivered entity into AC was approx. 6–12 mm^19,20^. In that study, we delivered large molecules of 2.8 kDa into a depth of 700 µm into AC within 2.5 hours. The present study demonstrated that a small molecule, methylene blue (320 Da) can be delivered to a similar depth of 600 µm as compared to C1 or C2, respectively, in a clinically relevant time frame of 15 minutes.

As suggested by our previous studies^18–21^, the delivery seen in this study may be explained by acoustic radiation forces, acoustic streaming, and non-deleterious cavitation^7,8,12,21^. Importantly, on average there were no major differences in light absorbance of C1 and C2 (Fig. 2A) suggesting that HIFU-induced modification of permeability in the time frame of less than hour is unlikely to explain the delivery. The sonication parameters were optimized to limit temperature increase to 3 °C suggesting a low thermal risk, supported by the low worst case thermal dose of 0.19 ± 0.30 and 0.17 ± 0.26 CEM43 (*N* = 5) for T1 and C1, respectively. Moreover, neither short-term mRNA expression levels nor cell viability were affected by HIFU. Therefore, damage to the tissue due to *e.g*. temperature is an unlikely explanation for delivery, although it may have minor effect^22,23^. However, C1 and C2 were incubated (Series 4000 TS 4057, Termaks, Bergen, Norway) at a temperature equivalent to T1 sample temperature suggesting that temperature as a main mechanism of delivery can be excluded in the experimental arrangement of the present study. Mechanical damage is also unlikely because of the moderate mechanical index of 1.8. Despite this, temporal behavior and reversibility of ultrasound-induced permeability enhancement in articular cartilage has not been studied, why permeability modification by HIFU cannot be fully excluded. As suggested for laser-ultrasonic drug delivery^21^, it is possible that the acoustic radiation force and acoustic streaming visualized in this study (Fig. 5) contribute towards high concentration of the delivered entity (MB) near the boundary, which could enhance molecule flux into AC^21^. Moreover, the positive charge of MB may be attracted by the negative fixed charge density inside AC, which in conjunction with HIFU induces or at least contributes to the delivery^21^.

The results of the present study suggest that the applied HIFU has no short-term influence on viability of mRNA expression of chondrocytes at 3.5 MPa peak-positive-pressure. This is in line with our previous study, which suggests that laser-ultrasound –induced shock waves with high peak-positive-pressure of 9.1 MPa (~3× the pressure applied in this study) did not affect chondrocyte viability or mRNA expression^21^. These studies, therefore, strengthen the understanding that HIU provides a safe drug delivery platform for development of new OA management strategies. However, it should be noted that this study investigated only short-term safety and, therefore, while unlikely, possible adverse effects in intermediate-term or long-term require further study. In addition, although active OA AC would be expected to be more permeable, this study was not set to address delivery of agents in osteoarthritic AC. A limitation of the study was the *in vitro* geometry, where sound propagation of the *in vivo* situation was not fully achieved; sound wave reflecting from the glass or aluminium sample holder at the back of the sample may have an effect on the results. We also limited the investigation to studying transport of molecules inside the tissue without identifying molecule micro-distributions *e.g*. near or within cells, which deserves attention in future studies.

While the technique presented in this study could be applied in open surgical settings, less invasive strategies would be strongly preferred. In a minimally invasive application, delivery of ultrasound actuation could be non-invasive and the delivered entity could potentially be injected intra-articularly. A minimally invasive approach could include an intra-articular applicator that would deposit both the ultrasound beam and the drug in or adjacent to the location of intended therapy (*e.g*. at a cartilage lesion).

To date, there are only limited studies suggesting delivery of drugs into AC by physical means. Electrostatic attraction between charged entities and fixed charges inside AC are known to enhance the delivery^24^. While not extensively investigated for AC as a means of drug delivery, electrophoresis^25^ or magnetophoresis could provide a way to localize drugs to intended locations on the joint surface. Since AC lesions can be focal^3^ and since a curative therapy to OA still remains to be discovered, there is an urge for more advanced drug delivery methods. Therefore, localized drug delivery by HIFU could potentially add value to the field.

To conclude, we demonstrated in this study that HIFU can deliver small molecules (320 Da) into AC without causing short-term concerns in viability or RNA expression in a clinically relevant time frame of 15 minutes. This approach is one candidate for a future clinical method for targeting drugs locally into AC.

## Materials and Methods

### Samples

Bovine joints (*N* = 11) from 11 animals were obtained from a local meat refinery (Lihakonttori Oy, Helsinki, Finland) within 6 days *post mortem*. This time window is sufficient to maintain viability of chondrocytes, which tolerate low-oxygen conditions. Osteochondral plugs (*N* = 10; diameter = 13 mm) with skeletal maturity were harvested, one plug per joint (Fig. 1) and excess subchondral bone was trimmed with a low-speed diamond saw (saw: Buehler Isomet, 11-1180-250; blade: 11–4256, Buehler) leaving 1–3 mm of bone beneath the AC tissue. Each osteochondral plug was cut with a scalpel and a hammer into four quadrants (Fig. 1). A set of 40 osteochondral quadrants from 10 joints were stored at −17 °C for later use in agent delivery experiments (Table 1). From one joint, one complete condyle was detached from one femur for stored at −17 °C for later use. For viability and RNA expression analyses bovine joints (*N* = 5) from 5 animals were obtained (Veijo Votkin Oy, Helsinki, Finland) within 60 hours *post mortem*. A set of 20 osteochondral quadrants from 5 joints (Fig. 1) were prepared and subjected to ultrasound experiments followed by viability and RNA expression analyses (Table 1).

### Experimental system

**Figure 6:**
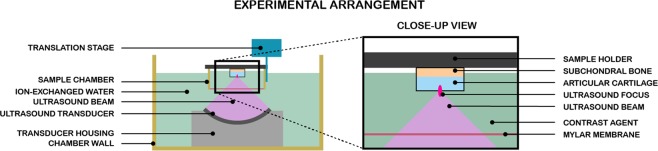
Experimental HIFU arrangement. The HIFU-treated samples were immersed in contrast agent solution, methylene blue in PBS, while sonicated. For osteochondral plugs and femoral condyle the holder plate was made of glass or aluminium, respectively.

A custom-made ultrasound system was built with a high-intensity focused ultrasound (HIFU) element driven with a function generator (model 33120 A, Hewlett Packard, Palo Alto, CA) and a power amplifier (model 500A100A, Amplifier Research). HIFU (*f* = 2.16 MHz, peak-positive-pressure = 3.5 MPa, mechanical index 1.8, pulse repetition frequency = 3.0 kHz, cycles per burst: 50, duty cycle: 7%, radius of curvature 53 mm, outer diameter = 36 mm) was used in sonication experiments (Fig. 6). The field was characterized at the ultrasound focus with a calibrated needle (0.2 mm needle hydrophone, Precision Acoustics, Dorchester, UK) after the wave had travelled through a 200 μm mylar membrane. The applied sonication parameters limited the maximum temperature increase in subchondral bone to 3 °C. Temperature of osteochondral plugs were detected during the sonication at the subchondral bone with a probe coupled thermally to the bone with a droplet of PBS. A custom-made thermometer system (probe K type, Fluke Corp., Everett, WA, USA; thermocouple amplifier AD595CQ, Analog Devices Inc., MA, USA; oscilloscope LeCroy 9310 A, Teledyne LeCroy Inc., Chestnut Ridge NY, USA; oscilloscope was connected to a PC via a GBIP connection) running on a Labview program (Labview Inc., Austin, TX, USA) was employed in the temperature measurement of sonicated samples. Back of the bone was selected based on a worst case principle; most of the heat energy is expected to be deposited in the bone. The temperature of the controls immersed in MB, while incubated, was measured from the MB solution (probe K type, Fluke Corp.; reader unit Tastotherm D 700, Gulton Inc., South Plainfield, NJ, USA).

### HIFU experiments

The samples were assigned to statistically dependent experimental groups (Fig. 1, Table 1).

*For agent delivery experiments*, methylene blue (MB; 0.005% w/v) in phosphate-buffered saline (PBS) was used as contrast agent. The four sample quadrants (*n* = 40) were thawed and subjected to following treatments:

(i) sonication with sample immersed in MB (*n* = 10; treatment group, T1);
(ii) first sonication in PBS, then sample immersion in MB (*n* = 10; control 1 group, C1);
(iii) no sonication, only sample immersion in MB (*n* = 10; control 2 group, C2); and
(iv) no sonication and no sample immersion in MB (*n* = 10; control 3 group, C3).

All four samples subjected to MB were immersed in MB solution for the same time (approx. 20 minutes for each set) to maintain comparability within each set. The sonication time was 15 minutes. During sonication, the articular surface faced the HIFU transducer, and the sample surface was positioned at the focus of the HIFU transducer. Laterally, the sonication was targeted at the center of the sample quadrant. After the treatment, AC was detached from the subchondral bone with a scalpel and immediately frozen to halt further diffusion of MB until assessment of delivery.

*For RNA and viability experiments*, the four quadrants (*n* = 20) were subjected to the following treatments:

(i) two quadrants were sonicated as described for drug delivery experiments while samples immersed in PBS (*n* = 10; treatment group, BT); and
(ii) the two remaining quadrants were not sonicated (*n* = 10; control group BC). Following the treatment, a cylindrical skin biopsy punch (diameter = 3 mm) and a scalpel were used to detach an AC cylinder from the center of the sample quadrant.

The rationale for having two paired samples per treatment was to secure sufficient amounts of tissue for reliable RNA and cell viability assessment.

### Assessment of delivery

Following treatments, samples assigned to agent delivery experiments were subjected to optical quantification of light absorbance at a wavelength (657 nm) equivalent to peak absorbance in MB (665 nm) as described in Nieminen *et al*.^21^: surface-to-deep tissue sections with 150 µm slice thickness were obtained and transmission of light was imaged at with Zeiss microscope (Stemi-C 2000, Carl Zeiss, Oberkochen, Germany) and an array of light-emitting diodes (light source: part number 148 LXZ1 – PA01, Philips LumiLeds Lighting Company LLC, San Jose, CA) with an emission maximum at 657 nm with a an optical diffuser (ground glass). The Napierian light absorbance was characterized depth-wise and the contribution of absorbance in native cartilage was minimized depth-wise by subtracting the absorbance in C3^26^ from depth-wise absorbances of T1, C1, and C2.

### Lateral localization of delivery

To demonstrate localized delivery in a full bovine condyle, the prepared condyle was positioned so that the articular surface faced the HIFU transducer. The chamber between the HIFU transducer and the condyle was filled with MB in PBS solution as described earlier. The sample surface was positioned perpendicular to the sound beam, at the focus of the HIFU transducer followed by 15 min sonication with parameters described previously. After sonication, the condyle was photographed followed by global contrast and brightness adjustment of the image.

### Detection of acoustic streaming

Acoustic streaming was detected under a custom-made Schlieren imaging system^27^. Syringe pump, syringe and tubing were used to make water through a cylindrical nozzle yielding a water stream with diameter 1.2 mm and a flow velocity of 1.4 cm/s.

### Cell viability

To evaluate the toxicity of the ultrasound exposure on the chondrocytes viability within AC, colorimetric assay of lactate dehydrogenase (LDH) activity in the cell culture supernatant after 24 h post sonication was employed. The supernatant media was screened for production of lactate using the Cytotoxicity Detection Kit (Roche Applied Sciences GmbH, Penzberg, Germany) per the manufacturer’s instructions. Supernatant media from control experiments were used as the control. Optical absorbance values at 492 nm were measured using a spectrophotometric microplate reader (FLUOstar Omega, BMG Labtech GmbH, Offenburg, Germany), with background media absorbance values subtracted.

### RNA extraction

The AC explants were lysed in a TRIzol buffer (Invitrogen) according to a Trizol protocol^28^. The lysed products were purified in a column with the RNeasy kit (Qiagen) following the manufacturer’s instructions. RNA concentrations were measured using a NanoDrop ND-1000 instrument (Thermo Fisher Scientific, Waltham, MA). The RNA integrity number (RIN) and 28 s/18 s ratio were estimated using the RNA 6000 Nano Assays on an Agilent 2100 Bioanalyzer (Agilent Technologies, CA). After RNA quality analysis, cDNA was synthesized using approximately 1–2 μg of total RNA with an iScript™cDNA Synthesis Kit (Bio-Rad Laboratories, Hercules, CA) in a 20 μl reaction volume.

### Droplet digital polymerase chain reaction (ddPCR)

Absolute mRNA expression levels were measured using droplet digital PCR (QX200 Droplet Digital PCR system, Bio-Rad Laboratories). Reactions were conducted in pre-defined volumes with 10 μl of ddPCR EvaGreen SuperMix, 2 μl of target gene primer, 8 μl nuclease free water, and 1 μl cDNA sample, according to manufacturer’s instructions. This was followed by sample loading into a droplet generator cartridge and addition of droplet generation oil (7 μl) into sample cartridge parallel wells. This was followed by individual droplet generation. Once the droplets were generated (40 μl) they were transferred into columns of a 96-well PCR plate and sealed with a pierceable foil in a PX1™ PCR Plate Sealer instrument (Bio-Rad). The sealed plate was then positioned in a T100 Thermal Cycler (Bio-Rad). This was followed by the sequence: 95 °C for 10 min, followed by 40 cycles of 94 °C for 30 s and 60 °C for 1 min, followed by 98 °C for 10 min. After PCR, the sealed plate was positioned in the droplet reader for detection of completed PCR reactions in the individual droplets. The data was visualized and analyzed with QuantaSoft software v1.7 (Bio-Rad), which provided the fraction of positive droplets and calculated the amount of templates per droplet. This was based on a Poisson distribution with a precision estimate of 95% confidence interval (CI) for every droplet. Based on results from negative control wells containing water (instead of RNA), thresholds for detection were manually set. Data was then exported as a CSV file and imported to GraphPad Prism version 7 software (GraphPad Software, La Jolla, CA, USA) for graphical representation. Primer sequences are provided in Table 2.

**Table 2:** Primer pairs applied in droplet digital polymerase chain reactions as adapted from Nieminen *et al*.^21^.

Gene	Protein name	Primer Sequence
*ACAN*	Aggrecan	Forward: 5′-CGA TAC CCC ATC TCC AAG GC-3′
		Reverse 3′-AGT GAT ATT TCG GGG CAG GG-5′
*COL2A1*	Collagen, type II, alpha 1	Forward: 5′-ACT GCC AGT GGG CAA CCA-3′
		Reverse 3′-GTC ACC ACG ATC ACC TCT GG-5′
*MMP1*	Matrix metalloproteinase 1 (MMP-1)	Forward: 5′-AAA TCC CAC TCA GCC AGT CG-3′
		Reverse 3′-CCC TGT CGG CAA CCT CAT AA-5′
*MMP3*	Matrix metalloproteinase 3 (MMP-1)	Forward: 5′-CAC TCA ACC GAA CGT GAA GC-3′
		Reverse 3′-GCT GAC TGC ATC GAA GGA CA-5′
*MMP9*	Matrix metalloproteinase 9 (MMP-9)	Forward: 5′-GAG ATG CCC ACT TCG ACG AT-3′
		Reverse 3′-GAG CGA CCC TCA AAG GTG AA-5′
*MMP13*	Matrix metalloproteinase 13 (MMP-13)	Forward: 5′-GTT GCT GCC CAT GAG TTT GG-3′
		Reverse 3′-TGT CTG GCG TTT TGG GAT GT-5′
*TGFB*	Transforming growth factor beta	Forward: 5′-AAT AGA GGG CTT TCG CCT CAG-3′
		Reverse 3′-AGC AGT AGT TGG TGT CCA GG-5′
*TIMP3*	Tissue inhibitor of metalloproteinases 3	Forward: 5′-GGC CGA GTC TAT GAT GGC AA-3′
		Reverse 3′-GTT TGG ACT GGT AGC CAG GG-5′
*TNF*	Tumor necrosis factor	Forward: 5′-GAA GAG CAG TCC CCA GGT G-3′
		Reverse 3′-GGG CTA CCG GCT TGT TAC TT-5′

### Statistical analyses

Groups T1, C1, and C2 were compared at each depth using a non-parametric Friedman test followed by a non-parametric Wilcoxon signed-rank *post-hoc* test for 2 related samples (SPSS v25.0.0.0, Chicago, IL, USA). The mRNA expression levels and viability of sonicated samples BT were compared against non-sonicated controls BC with non-parametric Wilcoxon signed-rank *post-hoc* test for two related samples.
